# FGF23-Mediated Activation of Local RAAS Promotes Cardiac Hypertrophy and Fibrosis

**DOI:** 10.3390/ijms20184634

**Published:** 2019-09-18

**Authors:** Ineke Böckmann, Jonas Lischka, Beatrice Richter, Jennifer Deppe, Anja Rahn, Dagmar-Christiane Fischer, Jörg Heineke, Dieter Haffner, Maren Leifheit-Nestler

**Affiliations:** 1 Department of Pediatric Kidney, Liver and Metabolic Diseases, Pediatric Research Center, Hannover Medical School, 30625 Hannover, Germany; 2 Division of Nephrology, Department of Medicine, The University of Alabama at Birmingham, Birmingham, AL 35294, USA; 3 Department of Pediatrics, Rostock University Medical Center, 18057 Rostock, Germany; 4 Department of Cardiology and Angiology, Experimental Cardiology, Hannover Medical School, 30625 Hannover, Germany; 5 Department of Cardiovascular Research, European Center for Angiosciences, German Center for Cardiovascular Research (DZHK), Medical Faculty Mannheim, Heidelberg University, 68167 Mannheim, Germany

**Keywords:** fibroblast growth factor 23, left ventricular hypertrophy, cardiac fibrosis, renin-angiotensin-aldosterone system, chronic kidney disease

## Abstract

Patients with chronic kidney disease (CKD) are prone to developing cardiac hypertrophy and fibrosis, which is associated with increased fibroblast growth factor 23 (FGF23) serum levels. Elevated circulating FGF23 was shown to induce left ventricular hypertrophy (LVH) via the calcineurin/NFAT pathway and contributed to cardiac fibrosis by stimulation of profibrotic factors. We hypothesized that FGF23 may also stimulate the local renin–angiotensin–aldosterone system (RAAS) in the heart, thereby further promoting the progression of FGF23-mediated cardiac pathologies. We evaluated LVH and fibrosis in association with cardiac FGF23 and activation of RAAS in heart tissue of 5/6 nephrectomized (5/6Nx) rats compared to sham-operated animals followed by in vitro studies with isolated neonatal rat ventricular myocytes and fibroblast (NRVM, NRCF), respectively. Uremic rats showed enhanced cardiomyocyte size and cardiac fibrosis compared with sham. The cardiac expression of *Fgf23* and RAAS genes were increased in 5/6Nx rats and correlated with the degree of cardiac fibrosis. In NRVM and NRCF, FGF23 stimulated the expression of RAAS genes and induced *Ngal* indicating mineralocorticoid receptor activation. The FGF23-mediated hypertrophic growth of NRVM and induction of NFAT target genes were attenuated by cyclosporine A, losartan and spironolactone. In NRCF, FGF23 induced *Tgfb* and *Ctgf*, which were suppressed by losartan and spironolactone, only. Our data suggest that FGF23-mediated activation of local RAAS in the heart promotes cardiac hypertrophy and fibrosis.

## 1. Introduction

Chronic kidney disease (CKD) is a global health issue [1] affecting over 850 million people worldwide according to the International Society of Nephrology (ISN). One of the major causes of death in this patient population are cardiovascular events [2,3], which are partially attributed to rising fibroblast growth factor 23 (FGF23) serum levels in CKD [4]. FGF23 is a phosphaturic hormone, mainly produced in osteocytes and osteoblasts [5]. Clinical studies in CKD patients showed a correlation of elevated FGF23 levels to left ventricular hypertrophy (LVH) [6,7]. Experimental research has further revealed that FGF23 directly induces LVH independently of its co-factor klotho by activation of FGF receptor 4 and subsequent calcineurin/nuclear factor of activated T cells (NFAT) signaling in vitro and in vivo [8,9]. Moreover, FGF23 is expressed by cardiomyocytes [10], and cardiac FGF23 expression is increased in uremic rats [11], transverse aortic constriction-operated mice [12] or mice after myocardial infarction [13] as well as in patients with CKD [14] and heart failure [15].

Activation of the renin–angiotensin–aldosterone system (RAAS) exacerbates renal failure [16] and contributes to LVH [17] in patients with CKD. When being less perfused, the juxtaglomerular apparatus secretes renin (REN), an enzyme that is responsible for cleaving liver-derived angiotensinogen (AGT) to angiotensin I (AngI), which is then further metabolized by angiotensin converting enzyme (ACE) to angiotensin II (AngII). Amongst other effects, AngII leads to peripheral vasoconstriction, activation of sympathetic nervous system and secretion of aldosterone from the adrenal glands by activating angiotensin II receptor type 1 (AT_1_R). Aldosterone enhances Na^+^ and H_2_O reabsorption in the distal tubules, effectively increasing blood pressure [18]. Apart from the systemic RAAS, various tissues, including the heart, show local RAAS expression [19]. Although the expression of RAAS in the heart remains controversial, Agt as well as Ren have been detected in both neonatal rat ventricular myocytes (NRVM) and cardiac fibroblasts (NRCF) [20], whereas ACE, AngI, AngII and AT_1_R have been shown in myocardial tissue [19]. Furthermore, experimental and clinical studies have revealed both AngII and aldosterone induce LVH in rodents and patients and lead to cardiac fibrosis in mice [21,22,23,24,25]. Recently, FGF23 has been linked to RAAS. On the one hand, AngII and aldosterone increase FGF23 serum levels in rodents [21,26] and AngII enhances, although to a lesser extent compared to the bone, cardiac specific FGF23 expression in wild-type mice [21]. On the other hand, FGF23 is discussed to activate RAAS by inhibiting renal expression of ACE2 [27], an enzyme converting AngII to the vasodilative and cardioprotective Ang 1–7 [28]. Additionally, FGF23-mediated reduction of 1,25-dihydroxy vitamin D_3_ (1,25(OH)_2_D_3_) levels [29] leading to the activation of renin, which is physiologically inhibited by active vitamin D [30]. In addition, we recently showed increased expression of *AGT* in myocardial tissue of CKD patients on dialysis and in cultured NRVM stimulated with FGF23 using fibrosis profiler PCR array analysis [31]. Although, the underlying molecular mechanisms are unknown, we hypothesized that FGF23-induced activation of the local RAAS contributes to cardiac pathologies in CKD.

Here, we investigated whether FGF23-mediated activation of endogenous cardiac RAAS contributes to cardiac hypertrophy and fibrosis by using the well-established 5/6 nephrectomy (5/6Nx) rat model of experimental uremia followed by in vitro analyses in NRVM and NRCF.

## 2. Results

### 2.1. Cardiac Hypertrophy and Left Ventricular (LV) Fibrosis Are Enhanced in Experimental Uremia and Associated with Increased FGF23 Synthesis in Heart and Bone

Cardiac hypertrophy and fibrosis are common comorbidities in CKD patients [32] and it is well-established that FGF23 directly promotes LVH via calcineurin/NFAT signaling activation in uremia [9,33]. Whether FGF23 contributes to the development of cardiac fibrosis is still controversial [31,34]. Here, we used 5/6Nx to induce CKD in rats and investigated LVH and LV fibrosis in association with FGF23. As published before [11], 5/6Nx rats showed increased heart weight to body weight ratio accompanied with enhanced cardiomyocyte size, enhanced mRNA expression of *Fgf23* in heart and bone tissue and significantly decreased phosphorylation of NFAT suggesting FGF23-mediated activation of calcineurin/NFAT pathway due to uremia (Table 1). Moreover, cardiac *Fgf23* expression significantly correlated with the cardiomyocyte cross-sectional area (*r* = 0.680; *p* = 0.011), while the expression of *Fgf23* in the bone just missed a statistically significant correlation with the cardiomyocyte size (*r* = 0.546; *p* = 0.051).

**Figure 1:**
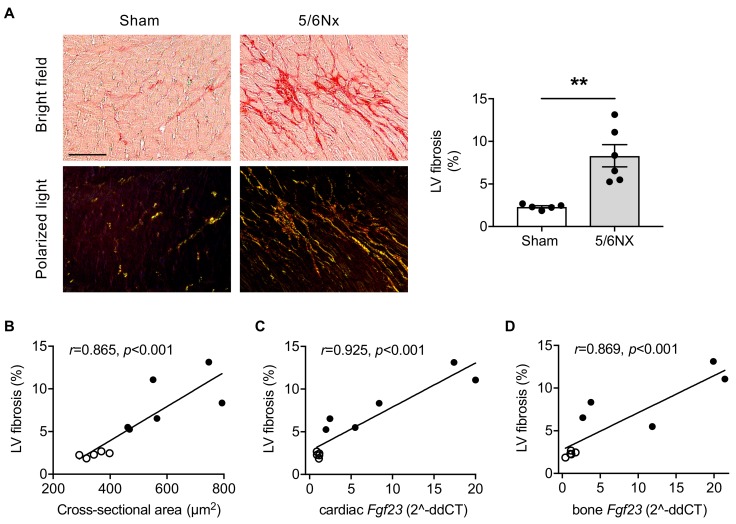
The 5/6 nephrectomized (5/6Nx) rats develop left ventricular (LV) fibrosis, which correlates with fibroblast growth factor 23 (*Fgf23*) expression. (**A**) Representative bright field and polarized light microscopy images of picrosirius red-stained myocardial tissue of 5/6Nx and sham-operated rats and quantification of interstitial collagen fiber deposition demonstrating increased LV fibrosis in 5/6Nx rats (scale bar, 50 µm). (**B–D**) Pearson’s correlations of LV fibrosis with cross-sectional area of cardiomyocytes, and cardiac and bone *Fgf23* mRNA expression as determined by quantitative real-time PCR using *Gapdh* as housekeeping gene. Clear dots, sham-operated rats; black dots, 5/6Nx rats. All values are shown as mean ± SEM; ** *p* < 0.01; *n* = 5–6 rats per group.

In addition to LVH, 5/6Nx rats developed severe LV fibrosis compared with sham as indicated by picrosirius red stained myocardial tissue and quantification of collagen matrix deposition (Figure 1A). The amount of LV fibrosis correlated with the cross-sectional area of cardiomyocytes (Figure 1B) and with both cardiac and bone *Fgf23* mRNA expression (Figure 1C,D).

### 2.2. Cardiac Expression of RAAS-Associated Genes is Increased in 5/6Nx Rats and Correlates with LV Fibrosis

**Figure 2:**
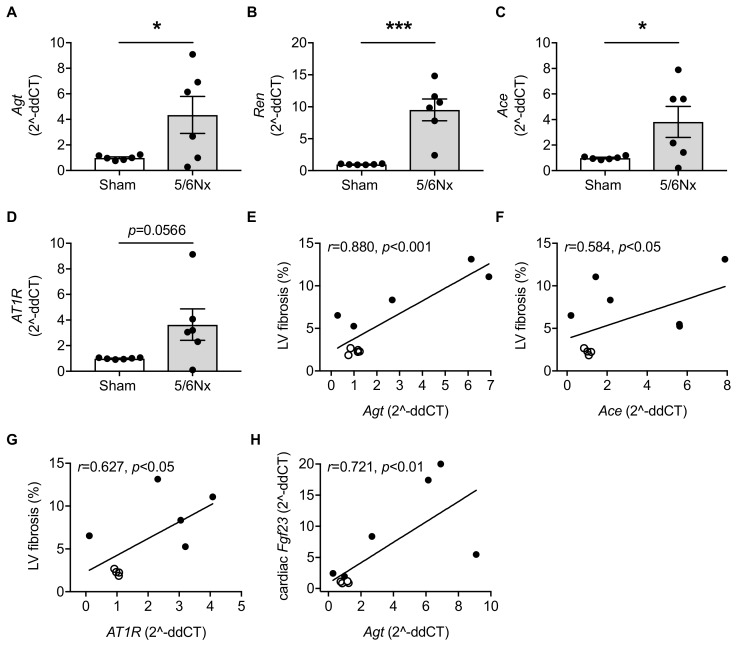
Here, 5/6 nephrectomy (5/6Nx) in rats induces cardiac expression of renin-angiotensin-aldosterone system (RAAS)-associated genes that correlates with left ventricular (LV) fibrosis. (**A–C**) Quantitative real-time PCR analyses in 5/6Nx rats show cardiac-specific induction of angiotensinogen (*Agt*), renin (*Ren*) and angiotensin converting enzyme (*Ace*), while (**D**) cardiac angiotensin II receptor type 1 (*AT1R*) mRNA expression is not significantly induced. (**E–G**) Pearson’s correlations of LV fibrosis with cardiac expression of *Agt*, *Ace* and *AT1R*, and (**H**) of cardiac *Fgf23* with *Agt* mRNA expression. Clear dots, sham-operated rats; black dots, 5/6Nx rats. All values are shown as mean ± SEM; * *p* < 0.05, *** *p* < 0.001; *n* = 5–6 rats per group.

Studies conducted in neonatal rat hearts and autopsied human hearts postulate the presence of endogenous RAAS in the heart [20,35], which contributes to cardiac hypertrophy and diastolic dysfunction [36,37]. Next, we investigated the local expression of RAAS-associated genes in heart tissue of 5/6Nx rats compared to sham and determined whether induction of uremia modulated the activation of RAAS. Cardiac-specific expression of *Agt*, *Ren*, *Ace* and *AT1R* were induced in 5/6Nx rats, although the latter did not reach statistical significance (Figure 2A–D). Remarkably, enhanced cardiac expression of *Agt*, *Ace* and *AT1R* correlated with the degree of LV fibrosis (Figure 2E–G), indicating the interaction of local RAAS activation and fibrotic remodeling in hearts of CKD rats. Interestingly, uremia-induced cardiac *Fgf23* significantly correlated with endogenous *Agt* mRNA expression (Figure 2H) as first parameter of the RAAS pathway. As we have shown previously, cardiomyocyte size, cardiac Fgf23 synthesis and expression of *Fgfr4* were negatively associated with pNFAT in 5/6Nx rats [11]. In the present study, LV fibrosis did not correlate with *Fgfr4* (*r* = 0.144, *p* = 0.346) and activation of NFAT (*r* = −0.359, *p* = 0.154), indicating that LV fibrosis in 5/6Nx rats was not mediated via FGF23-activated FGFR4/calcineurin/NFAT pathway. Taken together, these in vivo results suggest a direct relationship between FGF23, local RAAS and the progression of LV fibrosis in experimental uremia.

### 2.3. FGF23 Activates Local RAAS in Cardiomyocytes and Cardiac Fibroblasts in vitro by Increasing Expression of Agt, Ren, Ace and Ngal

**Figure 3:**
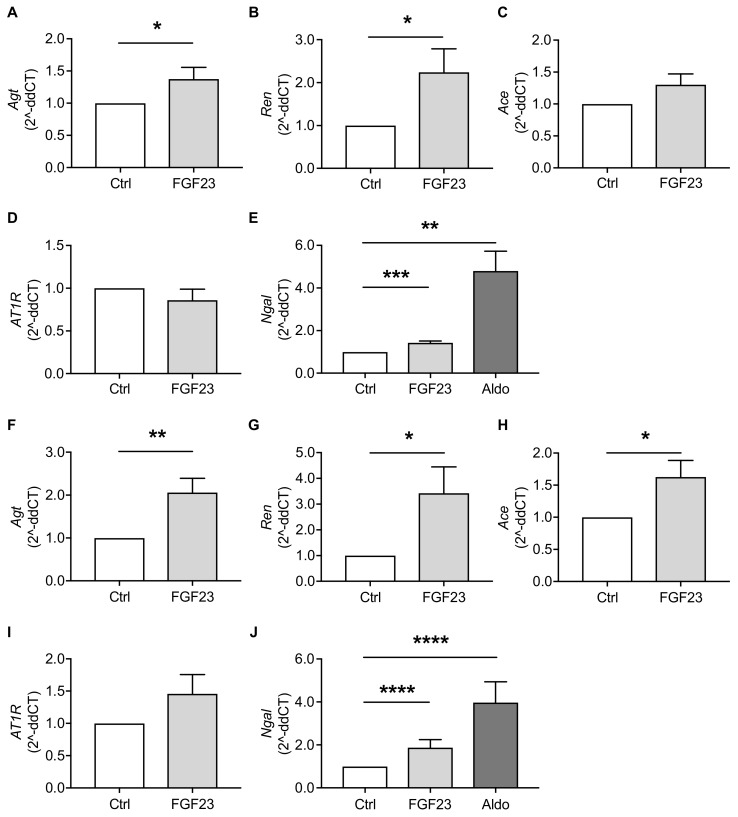
FGF23 induces RAAS-associated genes in neonatal rat ventricular myocytes (NRVM) and cardiac fibroblasts (NRCF) in vitro as shown by quantitative real-time PCR. (**A–D**) In NRVM, FGF23 treatment increases the mRNA expression of angiotensinogen (*Agt*) and renin (*Ren*), whereas the effects of FGF23 on angiotensin converting enzyme (*Ace*) and angiotensin II receptor type 1 (*AT1R*) expression levels are not statistically significant. (**E**) FGF23 and aldosterone (Aldo) induce the mRNA expression of neutrophil gelatinase-associated lipocalin (*Ngal*) in NRVM. (**F–I**) In NRCF, *Agt*, *Ace* and *Ren* mRNA expressions are enhanced after FGF23 treatment, but FGF23 does not significantly induce *AT1R*. (**J**) *Ngal* mRNA expression is elevated upon FGF23 and aldosterone stimulation. All values are shown as mean ± SEM; * *p* < 0.05, ** *p* < 0.01, *** *p* < 0.001, **** *p* < 0.0001 versus control; *n* = 6–8 independent cell isolations.

To verify the activation of RAAS, we analyzed the impact of FGF23 on RAAS-associated genes in NRVM and NRCF individually. *Agt*, *Ren*, *Ace* and *AT1R* were all clearly expressed in both cardiac cell types. In NRVM, FGF23 stimulation induced *Agt* and *Ren* (Figure 3A,B) and slightly increased *Ace* expression, although the latter did not reach statistical significance (*p* = 0.0705) (Figure 3C). On the contrary, FGF23 treatment did not alter mRNA expression levels of *AT1R* in NRVM (Figure 3D). However, as an indirect approach to determine FGF23-mediated production of AngII and aldosterone, we quantified the expression of neutrophil gelatinase-associated lipocalin (*Ngal*), which is a direct marker for mineralocorticoid receptor (MR) activation [38]. In NRVM, *Ngal* was induced in response to treatments with FGF23 and aldosterone as positive control (Figure 3E).

In NRCF, *Agt*, *Ren* and *Ace* were markedly induced by FGF23 (Figure 3F–H). A potential influence of FGF23 on *AT1R* mRNA expression was not statistically significant (*p* = 0.1801; Figure 3I). Nevertheless, consistent with FGF23-mediated effects observed in NRVM, *Ngal* expression in NRCF was stimulated by FGF23 and aldosterone (Figure 3J). In summary, these data affirmed our recent findings on FGF23-enhanced cardiac expression of *Agt* [31] and suggest that FGF23 further stimulates RAAS by induction of *Ren* and *Ace* in cardiomyocytes and cardiac fibroblasts in vitro. Moreover, the elevation of *Ngal* mRNA levels points towards an increased aldosterone activity because of FGF23 treatment in both cardiac cell types.

### 2.4. FGF23-Induced Hypertrophy in Cultured Cardiomyocytes is Prevented by Cyclosporine A, Losartan and Spironolactone

**Figure 4:**
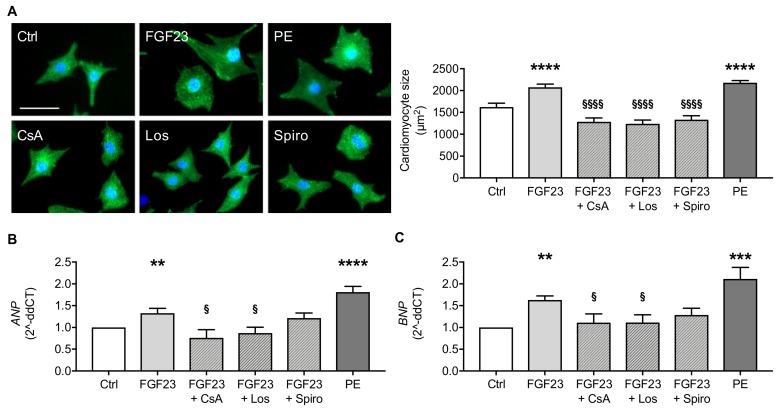
FGF23-induced hypertrophy in cultured NRVM is prevented by cyclosporine A (CsA), losartan (Los) and spironolactone (Spiro). (**A**) Representative immunofluorescent images of NRVM stained with sarcomeric α-actinin (green) and DAPI (blue) (scale bar, 50 µm), and quantification of NRVM size show significant induction of hypertrophic growth after treatment with FGF23, which is ameliorated by co-treatment with CsA, Los and Spiro. Phenylephrine (PE) served as positive control. (**B**,**C**) Quantitative real-time PCR analyses reveal FGF23-mediated increase of mRNA expression of prohypertrophic markers *ANP* and *BNP,* which is inhibited by CsA, Los and Spiro. *Gapdh* served as housekeeping gene. All values are shown as mean ± SEM; ** *p* < 0.01, *** *p* < 0.001, **** *p* < 0.0001 versus control; ^§^ *p* < 0.05, ^§§§§^ *p* < 0.0001 versus FGF23; *n* = 5–9 independent cell isolations.

In order to distinguish between FGF23-mediated cardiac hypertrophy via induction of calcineurin/NFAT pathway or activation of RAAS, we stimulated NRVM with FGF23 in the presence and absence of calcineurin inhibitor cyclosporine A (CsA), angiotensin receptor blocker losartan (Los) and steroidal mineralocorticoid receptor (MR) antagonist spironolactone (Spiro) followed by quantification of cardiomyocyte cell size as demonstrated by fluorescent-labeled sarcomeric α-actinin staining and expression of prohypertrophic NFAT target genes. In line with previous experimental studies [8,9], FGF23 stimulation resulted in hypertrophic growth of NRVM and induction of atrial natriuretic peptide (*ANP*) and brain natriuretic peptide (*BNP*), well-established markers for cardiac hypertrophy [39], which were inhibited by co-treatment with CsA (Figure 4A–C). Importantly, blockade of AT_1_R by Los and MR by Spiro also prevented FGF23-mediated cardiomyocyte cell hypertrophy to the same extent as CsA (Figure 4A). Moreover, the induction of *ANP* and *BNP* by FGF23 was clearly suppressed in the presence of Los, whereas inhibition of hypertrophic genes by co-treatment with Spiro did not reach statistical significance (Figure 4B,C). Phenylephrine served as positive control for cardiac hypertrophy and induction of *ANP* and *BNP* [40]. Taken together, besides the established activation of calcineurin/NFAT pathway by FGF23, our data suggest a RAAS-mediated signaling cascade of FGF23-induced cardiac hypertrophy.

### 2.5. FGF23-Mediated Induction of Profibrotic Markers TGFβ, CTGF and Collagen 1 is Attenuated by Inhibition of AT_1_R and MR

**Figure 5:**
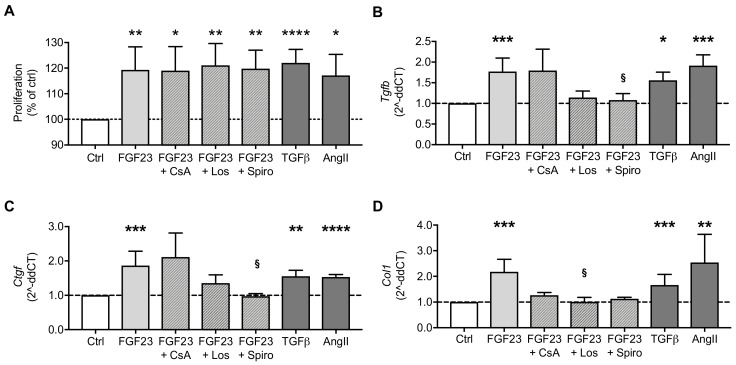
FGF23 stimulates the proliferation of NRCF and induces the expression of the profibrotic markers collagen 1 (*Col1*), transforming growth factor beta 1 (*Tgfb*) and connective tissue growth factor (*Ctgf*). (**A**) The proliferation of NRCF increases in response to stimulation with FGF23, which was not inhibited in the presence of cyclosporine A (CsA), losartan (Los) or spironolactone (Spiro) as demonstrated by MTS-based proliferation assay. (**B–D**) The mRNA expression of *Tgfb, Ctgf* and *Col1* in NRCF is increased upon FGF23 stimulation as measured by quantitative real-time PCR. Co-treatment with Los and Spiro inhibits the induction of fibrotic markers by FGF23, while CsA has lesser effects. TGFβ and angiotensin II (AngII) served as positive controls. Horizontal dotted lines represent the level of controls. All values are shown as mean ± SEM; * *p* < 0.05, ** *p* < 0.01, *** *p* < 0.001, **** *p* < 0.0001 versus control; ^§^ *p* < 0.05 versus FGF23; *n* = 5–9 independent cell isolations.

Due to stress stimuli or cardiac injury, AngII and aldosterone are induced and promote the differentiation of cardiac fibroblasts into activated myofibroblasts [23,41]. Myofibroblasts proliferate and migrate within the cardiac tissue and induce collagen synthesis and extracellular matrix (ECM) remodeling, which finally results in fibrosis and cardiac dysfunction. On the molecular level, AngII binds to AT_1_R and induces the expression of ECM proteins, transforming growth factor β (TGFβ) and endothelin-1 among others via activation of different signaling mediators. TGFβ binds to a heterodimer complex of TGFβ receptors and the fibrotic effect is further enhanced [42]. By using fibrosis PCR array analysis, we recently showed that FGF23 stimulated above-mentioned fibrosis-associated factors in NRVM and NRCF [31]. However, it is unclear whether FGF23 induces cardiac fibrosis via calcineurin/NFAT signaling [43] or through activation of RAAS. Thus, we stimulated NRCF with FGF23 in the presence or absence of CsA, Los and Spiro and investigated proliferation, TGFβ and connective tissue growth factor (CTGF) induction as key mediators of fibrotic remodeling [42] and collagen (COL) production as functional endpoint of fibrosis [44]. TGFβ and AngII served as positive controls for induction of profibrotic markers [23,42] as well as for proliferation of NRCF [45,46]. FGF23 promoted the proliferation of NRCF to the same extent as TGFβ and AngII but FGF23-mediated proliferation was neither suppressed by inhibition of calcineurin nor by Los or Spiro co-treatment (Figure 5A). FGF23 enhanced *Tgfb* and *Ctgf* mRNA levels, which were reduced by pharmacological blockade of AT_1_R and MR (Figure 5B,C). However, only the latter reached levels of statistical significance. Interestingly, FGF23-stimulated *Col1* expression was attenuated by inhibition of calcineurin through CsA and blockade of RAAS by co-treatment with Los and Spiro (Figure 5D). These data indicate that FGF23 mediates the induction of fibrotic remodeling mediators mainly through the activation of RAAS followed by AngII and aldosterone synthesis.

## 3. Discussion

One of the main causes of CVD in CKD are the increased circulating FGF23 levels, which were shown to induce LVH via the calcineurin/NFAT pathway [9,33] and to contribute to cardiac fibrosis through the activation of profibrotic factors [31,34]. In addition, both clinical and experimental research has shown increased activation of systemic RAAS in CKD [47,48], e.g., due to increased sympathetic activity [49] and reduced glomerular filtration rate leading to increased blood volume [50], which might further promote LVH and fibrosis in these patients. Whether a stimulated local RAAS in the heart contributes to CVD in CKD has not yet been extensively investigated. Although, FGF23 is discussed to stimulate RAAS [27,29,31], which may be an alternative mechanism for the progression of FGF23-mediated cardiac pathologies, the underlying molecular mechanisms are not well understood, yet. The present study showed that experimental uremia and FGF23 stimulation induced the activation of cardiac RAAS promoting LVH and cardiac fibrosis, which could be pharmacologically blocked by losartan and spironolactone in vitro. In particular, we showed considerable induction of RAAS genes in heart tissue of 5/6Nx rats, which correlated with the degree of LV fibrosis. Thus, local RAAS activation potentially contributes to the progression of CVD as comorbidity in renal failure. The local induction of RAAS genes in the heart supported recently published data from Beraldo et al. showing enhanced expression of *AGT*, *ACE*, and *AT1R* in heart tissue of 5/6Nx rats [51]. However, the expression levels of all three genes were higher in the present study which might be due to a longer CKD progression (twelve weeks versus eight weeks) and a different 5/6 renal ablation (two-step versus single-step surgery). Our data further complies with findings on increased *Agt* formation and elevated levels of local AngII in hearts of uremic rats and heart failure patients [52,53]. In the present study, cardiac *Fgf23* correlated with *Agt* expression in experimental uremia and FGF23 increased expression of RAAS-associated genes in both NRVM and NRCF in vitro, confirming previous work of our research group [31]. In NRVM and NRCF, FGF23 stimulated *Ren* mRNA most likely by direct effects. However, the increased *Ren* expression in myocardial tissue of 5/6Nx rats might also result indirectly from FGF23-mediated inhibition of active vitamin D synthesis in CKD. FGF23 decreases renal 1,25(OH)_2_D_3_ levels by both promoting its degradation and downregulating its synthesis [29], whereas active vitamin D in turn has been shown to decrease *Ren* mRNA levels in kidney-derived cells in vitro [30], and specifically in the heart of vitamin D receptor knockout mice [54]. Moreover, Freundlich et al. attributed cardioprotective effects of the vitamin D analog paricalcitol to the suppression of cardiac *Agt* and *Ren* expression in 5/6Nx rats [52]. In consideration of FGF23-induced downregulation of vitamin D [29], these findings suggest another molecular mechanism of FGF23-triggered myocardial RAAS activation that promotes LVH. Nevertheless, it has to be further proven whether the FGF23-mediated induction of *Ren* in cardiac cells results from a local downregulation of 1,25(OH)_2_D_3_ synthesis.

Although AT_1_R was not regulated on transcriptional level in NRVM and NRCF, *Ngal,* as target of the MR and indirect surrogate marker for aldosterone activity, was significantly induced upon FGF23 stimulation in vitro. These data suggest that FGF23 stimulated the activation of RAAS and thereby, resulting in enhanced AngII and aldosterone synthesis to further trigger cardiac hypertrophy and fibrosis. However, Ngal per se also correlates with the progression of CVD in CKD patients [55,56] and mediates cardiac fibrosis in mice after myocardial ischemia [57]. The present study supports these findings by demonstrating a protective role of MR inhibition by spironolactone on FGF23-mediated hypertrophic growth of NRVM, induction of prohypertrophic genes and fibrosis-associated factors TGFβ, CTGF and collagen I.

Multiple in vivo and in vitro studies demonstrated that FGF23-induced LVH is mediated by FGFR4 and successive phospholipase Cγ/calcineurin/NFAT signaling [8,9,14]. This is further consolidated by the present data on hypertrophic growth of NRVM and induction of fetal NFAT target genes *ANP* and *BNP* in response to FGF23 treatment. Furthermore, our findings confirm that FGF23-induced hypertrophy is prevented by pharmacological inhibition of calcineurin in vitro. It was shown in mice and patients that AngII and aldosterone induce LVH through inflammatory processes, which then is attenuated by treatment with angiotensin receptor blocker (ARB) and mineralocorticoid receptor antagonists (MRA), respectively [22,23,25]. Mhatre et al. recently showed that FGF23 and AngII induce a Ca^2+^ release from nucleoplasmic Ca^2+^ stores in NRVM, which was associated with cellular hypertrophy [58]. In addition, they demonstrated the inhibition of FGF23-induced cellular hypertrophy by losartan. Furthermore, FGF23 stimulation induced intracellular synthesis and secretion of AngII in NRVM [58], indicating that AngII mediates FGF23-triggered hypertrophy in vitro. In addition to the findings of Mhatre et al., inhibition of MR and ARB, in the present study, exerted similar protective effects on FGF23-mediated NRVM hypertrophy on cellular and gene expression level. Beyond that, our findings further point to an interaction of FGF23 and activated RAAS in cardiac fibrosis.

Pathological LVH is accompanied by cardiac fibrosis, which is enhanced in patients with CKD [59]. Accordingly, experimental uremia induced by 5/6Nx in rats enhanced LV fibrosis, which correlated with cardiomyocyte size. FGF23-mediated LVH is well documented [8,9,11,14,33], whereas the association to cardiac fibrosis is controversial [31,34]. Recently, FGF23 has been found to promote fibrosis in injury-primed renal fibroblasts through activation of both TGFβ/Smad and FGFR4/PLCγ/calcineurin/NFAT signaling [60,61], and to cause cardiac fibrosis by upregulating β-catenin and TGFβ in vitro and after myocardial infarction in vivo [34]. Using a fibrosis RT-PCR array in myocardial tissue of CKD patients on dialysis, *AGT*, TGFβ receptor/Smad complexes, *TGFB* and *CTGF* were significantly enhanced compared to controls, accompanied with more pronounced interstitial collagen fiber deposition, whereas in NRCF stimulated with FGF23 show no alteration of, *Agt*, *Tgfb* and *Ctgf*. However, FGF23 induced TGFβ receptor/Smad complexes, collagen synthesis and extracellular matrix remodeling factors in vitro [31]. Whether FGF23 induces profibrotic processes in the heart and whether this is mediated via calcineurin/NFAT or additional pathways still remains unclear. Here, enhanced LV fibrosis correlated with both bone and cardiac-specific *Fgf23* expression, implying a causal relation, which was further supported by in vitro findings on FGF23-induced *Tgfb*, *Ctgf* and *Col1* expression and proliferation of NRCF. Although, *Col1* was slightly reduced, *Tgfb* and *Ctgf* were not suppressed by inhibition of calcineurin through CsA in NRCF, which is in contrast to FGF23-triggered fibrosis in the kidney via calcineurin/NFAT [61]. Alternatively, our data suggest FGF23 promotes cardiac fibrosis by activating local RAAS, since LV fibrosis of 5/6Nx rats significantly correlated with enhanced cardiac mRNA expression levels of *Agt*, *Ace* and *AT1R*, and FGF23-induced fibrotic processes were prevented by co-treatment with Los and Spiro in NRCF. Both AngII and aldosterone cause myocardial fibrosis in mice, which may be attenuated by ARB and MRA, respectively [22,23,41,50]. 

The inductions of *AGT*, *REN, ACE* and *AT1R* on transcriptional level were shown to be mediated by the transcription factor activator protein 1 (AP-1), e.g., via activation of ERK1/2 [62,63,64,65]. In addition, the induction of AGT was further linked to activation of signal transducer and activator of transcription 3 (Stat3), glucocorticoid receptor, and CCAAT/enhancer-binding protein beta (CEBP-β) [66]. Interestingly, in atrial fibrillation, FGF23 mediated profibrotic response via Stat3 and Smad pathways in cardiac fibroblasts [67], and CEBP-β was significantly upregulated in myocardial tissue of patients on dialysis, which showed enhanced LVH and LV fibrosis [31]. Since FGF23 directly induced genes of RAAS in NRVM and NRCF, specific signaling mediators and transcription factors should be investigated in future studies.

One limitation of the present study is the associative nature of the in vivo data. Moreover, the removal of one kidney and the excision of 2/3 of the other kidney cause a large amount of kidney bleeding and infection, which on the one hand, increase the postoperative mortality of the animals, and on the other hand, can induce side effects such as inflammation, which might affect the investigated results. However, the 5/6Nx rat model of CKD is a well-established model of experimental uremia and widely used for several decades. Thereby it was shown that 5/6Nx in rodents shares features with the progressioin of human CKD, e.g., progressive loss of glomerular filtration rate, glomerulosclerosis, tubulointerstitial fibrosis, and proteinuria [68]. Thus, it is an excellent animal model for the translation to human disease, because 5/6Nx to rats and mice is a good stimulation of renal failure after loss of kidney function in humans [69,70].

Taking all results into account, our findings elucidate clinically observed interactions of elevated FGF23 levels and efficacy of RAAS inhibitors. In the Prevention of Events with Angiogensin Converting Enzyme Inhibition (PEACE) trial, FGF23 levels were associated with increased risk of cardiovascular death or heart failure even when adjusted for renal function and established cardiovascular markers, and, intriguingly, ACE inhibition by trandolapril significantly reduced these risks, but only for patients in the top quartile of FGF23 plasma concentrations [71]. These insights in patients with stable ischemic heart disease were also noticed in heart failure patients, who only benefited from ACE inhibition in case of elevated FGF23 levels [72].

In summary, we demonstrated an activation of endogenous cardiac RAAS in experimental uremia correlating with the progression of LV fibrosis and in response to FGF23 treatment in cardiomyocytes and cardiac fibroblasts in vitro. Our data confirm that FGF23-induced hypertrophic growth of cardiomyocytes is mediated by calcineurin/NFAT signaling and support the hypothesis that FGF23-mediated activation of local RAAS in the heart promotes cardiac hypertrophy and fibrosis in vitro and in vivo. These findings may at least partly explain why increased FGF23 plasma levels are associated with a better response to ACE inhibitor therapy in stable ischemic heart disease and chronic systolic heart failure [71,72].

## 4. Materials and Methods

### 4.1. Animal Experiments

The 5/6 nephrectomy (5/6Nx) and sham operation was performed in each *n* = 6 male Sprague Dawley rats as described previously [73]. Twelve weeks after surgery, animal weights were recorded, hearts were isolated and weighted, cardiac mid-chamber sections were fixed in 4% paraformaldehyde followed by paraffin-embedding for histological analysis, and residual cardiac tissue was snap-frozen in liquid nitrogen for molecular analysis. All experimental procedures were performed in accordance with the national animal protection guidelines from Directive 2010/63/EU of the European Parliament on the protection of animals used for scientific purposes, and approved by the Landesamt für Landwirtschaft, Lebensmittelsicherheit und Fischerei (LALLF) Mecklenburg-Vorpommern (LALLF M-V/TSD/7221.3-1.1-024/06). The description of the rat cohort and the determination of cardiac hypertrophy and FGF23/FGFR4 signaling pathway inducing LVH was published before [11].

### 4.2. Picrosirius Red Staining and Quantification of Myocardial Fibrosis

After deparaffinization in xylene followed by hydration in descending alcohol series, three µm thick cardiac mid-chamber sections were incubated in picrosirius red solution in 1.2% picric acid. For determination of cardiac fibrosis, five pictures per rat were taken using a 20× objective on a Zeiss Axio Observer Z1 microscope (Carl Zeiss Microscopy, Goettingen, Germany) and quantification of interstitial collagen fibers were performed with Image J software [74,75].

### 4.3. Isolation and Culture of Neonatal Rat Ventricular Myocytes (NRVM) and Cardiac Fibroblasts (NRCF)

Cells were isolated from one-day-old neonatal Sprague-Dawley rats by Percoll gradient as described previously [76]. In brief, hearts were extracted and digested in collagenase II (Worthington Biochemical, Lakewood, NJ, USA) and pancreatin (Sigma-Aldrich, Taufkirchen, Germany) solution. Cells were then separated into an upper phase of NRCF and a lower phase of NRVM by centrifugation in Percoll gradient. Then 6 × 10^5^ NRVM were seeded on 6 cm cell culture plates coated with 0.5% gelatin in NRVM plating medium including DMEM (Biochrom, Berlin, Germany) with 20% M199 (Pantech Biosolutions, Ruggel, Liechtenstein), 5% fetal bovine serum (FBS) (Serana Europe, Pessin, Germany) and 10% horse serum. Likewise, 6 × 10^5^ NRCF were seeded on 6 cm cell culture plates using NRCF plating medium (DMEM with 10% FBS). NRVM were used on the second day after isolation and NRCF were used from passage 1–2.

### 4.4. Stimulation of Isolated NRVM and NRCF

NRVM and NRCF were starved overnight in DMEM with 20% M199 and DMEM with 1% FBS, respectively. The next day, NRVM and NRCF were stimulated for 48 h with recombinant human FGF23 (NRVM, 100 ng/mL; NRCF, 10 ng/mL; R&D Systems, Wiesbaden, Germany) alone and in combination with 100 nM cyclosporine A (CsA), 1 µM losartan (Los) or 10 µM spironolactone (Spiro). Angiotensin II (AngII) at 100 nM, aldosterone (Aldo) at 100 nM, transforming growth factor β1 (TGFβ) at 0.5 ng/mL, and phenylephrine (PE) at 20 µM (all from Bio-Techne/Tocris, Wiesbaden, Germany) served as positive controls.

### 4.5. Immunofluorescence Staining and Morphometry of Cultured NRVM

For immunofluorescence-based quantification of NRVM size, cells were fixed with 4% paraformaldehyde in DPBS and permeabilized by 0.25% Triton X-100. Cells were incubated with mouse monoclonal antibody against sarcomeric α-actinin at 3 µg/mL (EA-53; Sigma-Aldrich, Taufkirchen, Germany) in 2.5% BSA in DPBS for 1 h followed by secondary Cy3-conjugated goat anti-mouse antibody at 2 µg/mL (Thermo Fisher Scientific, Bremen, Germany) in 2.5% BSA in DPBS for 30 min. For visualization of nuclei, NRVM were incubated with 4’,6-diamidino-2-phenylindole at 200 ng/mL (DAPI; Sigma-Aldrich, Taufkirchen, Germany) in DPBS for 12 min. Images of NRVM were taken on a Zeiss Axio Observer Z1 microscope (Carl Zeiss Microscopy, Goettingen, Germany) with a 20× objective. Average cardiomyocyte cell size was quantified by measuring at least 100 cells per group using Carl Zeiss Zen software.

### 4.6. RNA Isolation, cDNA Synthesis and Quantitative Real-Time PCR Analysis

For total RNA isolation from cells and rat hearts, the RNeasy Mini Kit was used according to the manufacturer’s protocol. 500 ng mRNA was transcribed into cDNA according to the data sheet of the QuantiTect Reverse Transcription Kit. Real-time polymerase chain reaction was run in triplets on a 7900 HT Fast RT-PCR System (Thermo Fisher Scientific, Bremen, Germany) using the QuantiFAST SYBR Green PCR Kit (all kits from Qiagen, Hilden, Germany). For rat primer sequences see Table 2. Relative gene expression levels of stimulated cells compared to control-treated cells were calculated by the use of the 2^−ddCT^ method using SDS Software v2.4 (Thermo Fisher Scientific, Bremen, Germany) and *Gapdh* served as a housekeeping gene.

### 4.7. Cell Proliferation Assay

Firstly, 5000 NRCF per well were seeded on a 96-well plate in quadruplicates per group. After stimulation for 48 h, 20 µL of MTS-based CellTiter 96 Aqueous One Solution Cell Proliferation Assay (Promega, Mannheim, Germany) was added per well and incubated for 4 h followed by measuring the absorbance at 490 nm with a Tecan M200 Infinite Pro 96-well plate reader (Tecan, Crailsheim, Germany). Values of absorbance were presented as percent proliferation of control cells. 

### 4.8. Statistical Analysis

Values are presented as mean ± standard error of mean (SEM) using GraphPad Prism software 7.00 (GraphPad Software, La Jolla, CA, USA). Gaussian’s distribution was analyzed by Shapiro Wilk or D’Agostino Pearson test and differences between groups were compared by the use of 2-tailed t-test or Mann–Whitney U test, respectively. Correlation analyses between parameter in sham and 5/6Nx rats were done by Pearson’s correlation using SPSS software version 25 (IBM, Armonk, NY, USA). *p* values <0.05 were considered as statistically significant.

## Figures and Tables

**Table 1:** Characteristics of sham and 5/6 nephrectomized (5/6Nx) rats.

Characteristics	Sham	5/6Nx	*p* Value
Number of rats (*n*)	6	6	
Heart weight/body weight (mg/g)	2.8 ± 0.1	3.7 ± 0.2	0.0005
Cardiomyocyte size (µm^2^)	344 ± 19	598 ± 57	0.0038
Cardiac *Fgf23* mRNA (2^−ddCT^)	1.00 ± 0.07	9.29 ± 3.15	0.0250
Bone *Fgf23* mRNA (2^−ddCT^)	1.00 ± 0.20	11.93 ± 3.91	0.0129
Cardiac *Fgfr1* mRNA (2^−ddCT^)	1.00 ± 0.06	7.91 ± 2.49	0.0196
Cardiac *Fgfr4* mRNA (2^−ddCT^)	1.00 ± 0.07	21.91 ± 10.56	0.0022
Cardiac pNFAT protein (fold change)	1.00	0.27 ± 0.18	0.0291

Values are presented as mean ± standard error of mean using data from a rat cohort published before [11].

**Table 2:** Rat primer sequences for quantitative real-time PCR analysis.

Gene	Forward (5′-3′)	Reverse (5′-3′)
*Gapdh*	ACTCCACGACATACTCAGCAC	CATCAACGACCCCTTCATT
*Agt*	CAGCACGACTTCCTGACTTGGAT	GGATGCTGTGA GAACCTCTCCCA
*Ren*	AGGATCAGTGCTGAATGGGGTGA	GGTTGTGAATCTCACAGGCAGTGT
*Ace*	TGCCTAGATCCCAAGGTGACTTTGA	CAACTTCATGGCATCTGCCAGCA
*AT1R*	GCTCTGCCACATTCCCTGAGTTA	CTTGGGGCAGTCATCTTGGATTCT
*Ngal*	GATGTTGTTATCCTTGAGGCCC	CACTGACTACGACCAGTTTGCC
*ANP*	AAATCCCGTATACAGTGCGG	GGAGGCATGACCTCATCTTC
*BNP*	CCAGAACAATCCACGATGC	TCGAAGTCTCTCCTGGATCC
*Col1*	AAGGGTCCTTCTGGAGAACC	TGGAGAGCCAGGGAGACCCA
*Tgfb*	GCAACAACGCAATCTATGAC	CCCTGTATTCCGTCTCCTT
*Ctgf*	CTGGAAGACACATTTGGCCC	CAGAAGGTATTGTCATTGGT
